# EGFR-AS1 Promotes Bladder Cancer Progression by Upregulating EGFR

**DOI:** 10.1155/2020/6665974

**Published:** 2020-12-22

**Authors:** Anbang Wang, Aimin Jiang, Xinxin Gan, Zheng Wang, Jinming Huang, Kai Dong, Bing Liu, Linhui Wang, Ming Chen

**Affiliations:** Department of Urology, Changzheng Hospital, Naval Medical University, Shanghai 200003, China

## Abstract

Long noncoding RNAs play an essential role in bladder cancer progression. The role of long noncoding RNA EGFR-AS1 in bladder cancer needs further study. We used clinical specimens to analyze the relationship between EGFR-AS1 and bladder cancer patients' characteristics. The functional experiments and mechanism studies were performed using qRT-PCR, transwell assay, survival analysis, and correlation analysis. We found that high expression of EGFR-AS1 was nearly related to aggressive bladder cancer and indicated poor prognosis for patients. The functional experiments in vivo and in vitro suggested that EGFR-AS1 promoted the proliferation and invasion of bladder cancer cells. Mechanically, EGFR-AS1 promoted the expression of EGFR by inhibiting the degradation of EGFR mRNA, thereby promoting the metastasis of bladder cancer. In addition, EGFR-AS1/EGFR may be involved in the immune-related pathways of bladder cancer. These studies indicate that the EGFR-AS1/EGFR pathway may be a potential diagnostic marker and therapeutic target for bladder cancer.

## 1. Introduction

Bladder cancer is one of the most common malignant tumors in the urinary system, ranking fourth and seventh for male and female tumor mortality globally, respectively. GLOBOCAN statistics estimate that there are 549,400 new cases and 199 thousand bladder cancer deaths in 2018 [1]. Whether the tumor has muscular infiltrating growth is the most important indicator to judge the prognosis. Muscle invasive bladder cancer (MIBC) possesses the characteristics of rapid progress, inescapable recurrence, uncomplicated distant metastasis, high malignancy, and high mortality [2]. Cisplatin combined with gemcitabine is the first-line treatment for metastatic bladder cancer. However, the objective response rate of chemotherapy is only 48%, the median disease progression time of patients is less than six months, and the overall survival time is 13.8 months. The low response rate and drug resistance of standard chemotherapy severely limit the effectiveness of chemotherapy. The targeted therapy that has emerged in recent years as a new treatment model with a definite curative effect and better tolerance of patients has already achieved initial results in treating malignant solid tumors. Current potential targets for bladder cancer include epidermal growth factor receptor, fibroblast growth factor receptor, mTOR signaling pathway, and immune checkpoint inhibitors [3, 4]. The effectiveness and specific mechanism of action of these targets still need to be further explored to improve the effectiveness of targeted therapy for bladder cancer. Therefore, it is imperative to study the potential mechanism of bladder cancer and find new targets for intervention.

Long noncoding RNA is a newly discovered noncoding RNA (lncRNA) of more than 200 nucleotides, studied in various diseases and biology [5]. lncRNA is associated with tumorigenesis and may become a new biomarker for tumor diagnosis, prognosis, and even targeted gene therapy. However, its primary mechanism still needs further study. By searching the TCGA and GEO databases in the early stage of the research group, long noncoding RNA EGFR-AS1 closely related to renal cancer metastasis was selected. Our research group found that EGFR-AS1 maintained the stability of EGFR mRNA by binding to the RNA binding protein HuR and promoted the proliferation and metastasis of renal cancer. The high expression of EGFR-AS1 was closely related to the poor prognosis of renal cancer patients [6]. Some recent studies have shown that EGFR-AS1 mainly plays a crucial role in cancer progression [7, 8]. Tan et al. found that EGFR-AS1 affected the sensitivity of squamous cell carcinoma to TKIs by regulating EGFR spliceosome [7]. The EGFR signaling pathway is also excessively activated in bladder cancer cells, which may directly promote the growth and metastasis of bladder cancer. Kim et al. showed that the EGFR expression level was a new prognostic indicator of disease progression for bladder cancer patients with local recurrence or metastatic MIBC [9]. However, the role of the EGFR-AS1/EGFR signaling pathway needs further research.

This study found that EGFR-AS1 was highly expressed in bladder cancer tissues and predicted poor prognosis of patients. Subsequent mechanism studies confirmed that EGFR-AS1 promoted the high expression of EGFR by maintaining its RNA stability, thereby promoting the progression of bladder cancer. Besides, EGFR-AS1 was involved in the immune-related pathways of bladder cancer. The results indicate that further studies are warranted to elucidate the complex pathway of EGFR-AS1 in bladder cancer.

## 2. Materials and Methods

### 2.1. Patient and Clinical Specimens

A total of 128 bladder cancer tissues and adjacent normal specimens were collected from postoperative bladder cancer patients at Changzheng Hospital, Naval Medical University. All tissues were frozen immediately after the operation and stored in -80-degree refrigerator. More than two pathologists confirmed all excised tissues. The pathological stage and grade of all tissues were evaluated according to the World Health Organization (WHO) criteria. All patients signed the informed consent for the study. The research project was approved by the Ethics Committee of Changzheng Hospital.

### 2.2. Immune Correlation Analysis

ImmLnc research and TIMER analysis were used to analyze the correlation between EGFR-AS1 and immune pathways. These two analysis tools are based on the network to analyze the difference of immune cell infiltration in different tumors (ImmLnc: http://bio-bigdata.hrbmu.edu.cn/ImmLnc/; TIMER: http://cistrome.dfci.harvard.edu/TIMER/).

### 2.3. Cell Culture and Transfection

The bladder cancer cell lines used in the experiment were all purchased from the American ATCC Cell Bank; 5637 and T24 cell lines were cultured in RPMI1640 medium (Gibco) containing 10% fetal bovine serum (HyClone). The cell culture conditions were 37°C, 5% CO_2_ saturated humidity incubator. We purchased the EGFR-AS1 knockdown and overexpressed lentiviruses (lv-shEGFR-AS1 and lv-oeEGFR-AS1) from Shanghai Heyuan Biotechnology and screened and verified them [6].

### 2.4. RNA Isolation and RT-PCR Analyses

Total RNA was extracted and separated using TRIzol reagent (Invitrogen, USA). The RT-PCR experiment was performed using the ABI 7900HT Fast Real-Time PCR System (Applied Biosystems, USA) and repeated three times. The expression level of RNA was calculated using *β*-actin as a standard internal parameter and 2^−*△△*Ct^ was calculated. The RNA primer sequences are as follows: EGFR-AS1 forward, 5′- CCATCACGTAGGCTTCCTGG-3′ and reverse, 5′- GCATTCATGCGTCTTCACCTG-3′ and EGFR forward, 5′- TGGTCAAGTGCTGGATGATAGA-3′ and reverse, 5′- ACGGTAGAAGTTGGAGTCTGTA-3′. RT-PCR experiments were performed in two bladder cancer cell lines.

### 2.5. Cell Proliferation Test

We used the MTT method to evaluate the proliferation ability of bladder cancer cells. The treated bladder cancer cells were planted in 96-well plates, with 2 × 10^3^ cells per well. After 5 days of cell culture, the cells were treated with MTT for 4 hours, and then, the absorbance of light with a wavelength of 490 nm in a microplate reader was compared with time. OD490 value here reflects the number of viable cells. Every cell proliferation experiment was repeated three times.

### 2.6. Transwell Test

We used the number of cells passing through Matrigel to evaluate the migration and invasion ability of bladder cancer cells. First of all, 3 × 10^5^ cells were seeded into 24-well plates. The cells were plated in serum-free medium, and the lower chamber contained the medium and 10% fetal bovine serum. After incubation for 24 hours, the cells that did not invade the pores were carefully wiped with a cotton swab. All cells migrating from the upper part of the filter to the lower part were fixed with 4% paraformaldehyde and stained with 1% crystal violet. Then, we count and image them (magnification ×100). These measurements were made at least three times.

### 2.7. Statistical Analysis

In this study, SPSS Statistics software version 18 (SPSS Inc., USA) and GraphPad Prism 6 software (GraphPad Software, Inc.) were used for statistical analysis. Depending on the type of data, suitable statistical methods were selected, including *t*-test, variable analysis, and chi-square test. Kaplan–Meier method with the log-rank test was used to compare the prognosis of patients with different EGFR-AS1 expressions. A *p* value of less than 0.05 on both sides indicates statistical significance.

## 3. Results

### 3.1. EGFR-AS1 Is Related to Cancer Progression and Participated in Immune Pathways of Bladder Cancer

**Figure 1:**
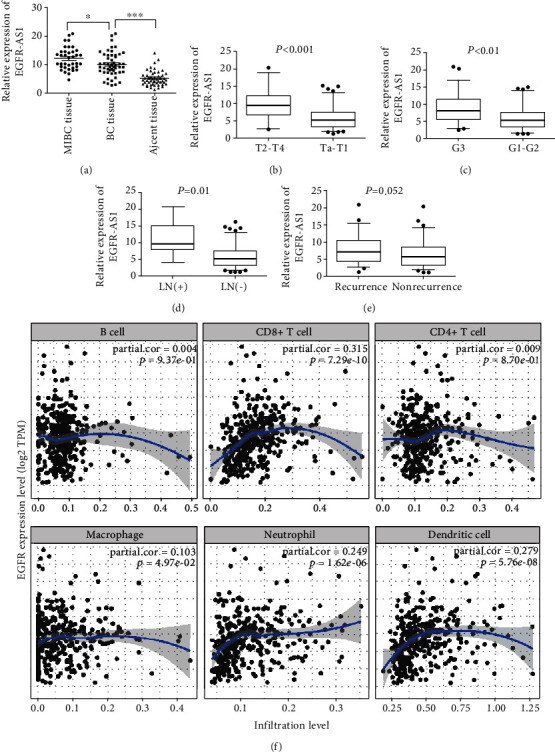
Expression of EGFR-AS1 in bladder cancer tissues of different groups. (a) EGFR-AS1 expression between cancer tissues and normal tissues was compared through RT-PCR analysis. ^∗^*p* < 0.05, ^∗∗^*p* < 0.01, and ^∗∗∗^*p* < 0.001 by Student's *t*-test. (b) EGFR-AS1 expression in different tumor stages (T2-T4, *n* = 36; Ta-T1, *n* = 92). *p* < 0.001 by the Mann–Whitney *U* test. (c) EGFR-AS1 expression in different tumor grades (G3, *n* = 56; G1-G2, *n* = 72). (d) EGFR-AS1 expression comparison between lymphatic metastasis positive cancer tissues and negative tissues. (e) EGFR-AS1 expression comparison between recurrent cancer tissues and no recurrence tissues. (f) The relationship between EGFR expression and infiltration level of immune cell in bladder cancer.

First, we analyzed the correlation between the EGFR-AS1 expression and clinical characteristics of bladder cancer patients. The qRT-PCR analysis found that the expression of EGFR-AS1 in bladder cancer tissues was higher than that in normal tissues. In particular, EGFR-AS1 was more expressed in muscle invasive bladder cancer (MIBC) tissues (Figure 1(a)). EGFR-AS1 was expressed higher in tumors > 4 cm than in tumors ≤ 4 cm (*p* < 0.001), in high-grade tumors than in low-grade tumors (*p* < 0.01), and in the lymph node metastasis group than in the no lymph node metastasis group (*p* < 0.01) (Figures 1(b)–1(e)).

According to the expression level of EGFR-AS1, 128 bladder cancer tissues were divided into the high-expression EGFR-AS1 group and low EGFR-AS1 group. High expression of EGFR-AS1 is closely related to larger tumor diameter, high grade, and lymphatic metastasis (Table 1). These results indicated that EGFR-AS1 was associated with aggressive clinical features of bladder cancer.

Immunotherapy has gradually shown a specific role in the treatment of advanced bladder cancer [10, 11]. We analyzed the correlation between EGFR-AS1 and immune pathways of bladder cancer. Using ImmLnc research, we found that EGFR-AS1 was strongly correlated with the TCR signaling pathway and cytokine receptors. In addition, the results showed that EGFR-AS1 was strongly related to CD8_T cell (Table 2). EGFR-AS1 can promote the expression of EGFR in urinary system cancer [6]. Using TIMER analysis, we identified that EGFR was significantly correlated with CD8+ T cell, neutrophil, and dendritic cell (Figure 1(f)). These results indicate that EGFR-AS1 may promote cancer progression by participating in the immune-related pathways of bladder cancer.

### 3.2. The Diagnostic and Prognostic Values of High EGFR-AS1 Expression in Bladder Cancer Patients

**Figure 2:**
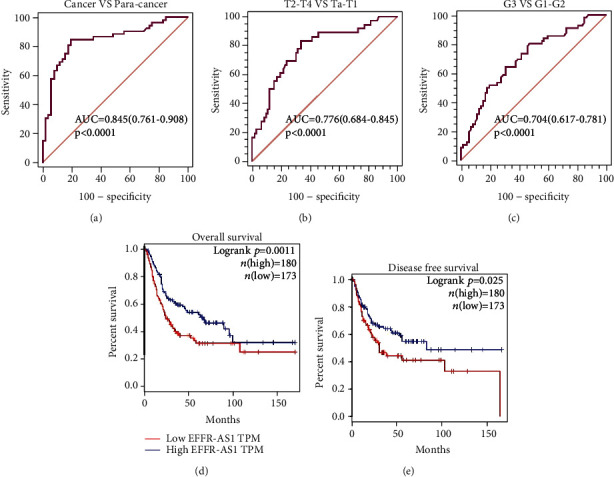
The diagnostic and prognostic values of the EGFR-AS1 expression in bladder cancer. (a) ROC curve analysis showed that EGFR-AS1 could efficiently distinguish bladder cancer from a normal individual. The area under curve (AUC) was 0.845 (*p* < 0.0001). ROC curve analysis towards the expression levels of EGFR-AS1 in bladder cancer subgroups against tumor stage (b) and tumor grade (c). Kaplan–Meier analysis of overall survival rate (d) and disease-free survival (e) of bladder cancer patients with high or low EGFR-AS1 expression in the TCGA database (*p* = 0.0011, *p* = 0.025).

We used the ROC curve to analyze the diagnostic value of the EGFR-AS1 expression in patients with bladder cancer. The study suggested that high EGFR-AS1 expression could statistically distinguish bladder cancer and adjacent tissues, and the area under the curve (AUC) was 0.845 (95% CI: 0.761-0.908, *p* < 0.0001) (Figure 2(a)). Similarly, high EGFR-AS1 expression could also discriminate the clinical stages (AUC = 0.776, *p* < 0.0001) and pathological grades (AUC = 0.704, *p* < 0.0001) (Figures 2(b) and 2(c)) of bladder cancer. The TCGA bladder cancer data analysis implied that the high expression of EGFR-AS1 was associated with poor prognosis of bladder cancer (Figures 2(d) and 2(e)). These results indicated that EGFR-AS1 may have diagnostic and prognostic values for bladder cancer patients.

### 3.3. EGFR-AS1 Facilitates the Proliferation and Invasion of Bladder Cancer Cells In Vitro

**Figure 3:**
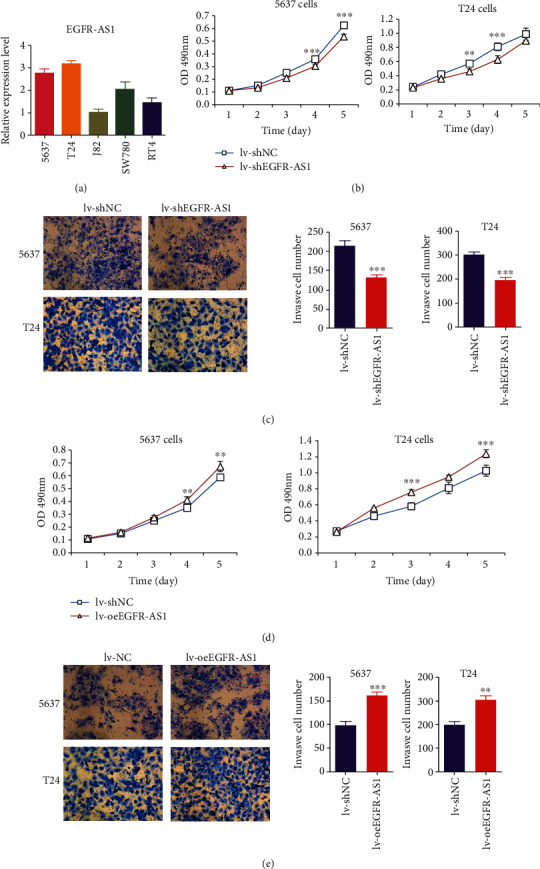
EGFR-AS1 facilitates bladder cancer cell proliferation and invasion in vitro. (a) The expression of EGFR-AS1 was detected in various bladder cancer cell lines. (b) Knocking down EGFR-AS1 inhibited 5637 and T24 cell proliferation by MTT assay. (c) Knocking down EGFR-AS1 suppressed the invasion capacity of 5637 and T24 cell using transwell assays. (A) The representative pictures of transwell assay, scale bar = 200 *μ*m. (B) The number of cells in six random fields (magnification, ×200). (d) Overexpressing EGFR-AS1 accelerated 5637 and T24 cell proliferation by MTT assay. (e) Overexpressing EGFR-AS1 enhancing the invasion ability of 5637 and T24 cells using transwell assay. (A) The representative pictures of transwell assay, scale bar = 200 *μ*m. (B) The number of cells in six random fields (magnification, ×200). ^∗^*p* < 0.05, ^∗∗^*p* < 0.01, and ^∗∗∗^*p* < 0.001 by Student's *t*-test.

The expression of EGFR-AS1 was detected in various bladder cancer cell lines. We found that EGFR-AS1 expressed higher levels in the T24 and 5637 cell lines (Figure 3(a)). Thus, we used the 5637 and T24 cell lines to construct EGFR-AS1 overexpression and interference cell lines (lv-oeEGFR-AS1, lv-shEGFR-AS1). MTT experiments indicated that knocking down EGFR-AS1 significantly inhibited the proliferative capacity of bladder cancer cell lines (Figure 3(b)). The transwell experiment showed that after downregulating EGFR-AS1, the invasion ability of cell lines significantly decreased (Figure 3(c)). Correspondingly, EGFR-AS1 overexpression promoted the proliferation and invasion capacity of bladder cancer cells (Figures 3(d) and 3(e)).

### 3.4. Knocking down EGFR-AS1 Inhibits Bladder Cancer Growth and Metastasis In Vivo

**Figure 4:**
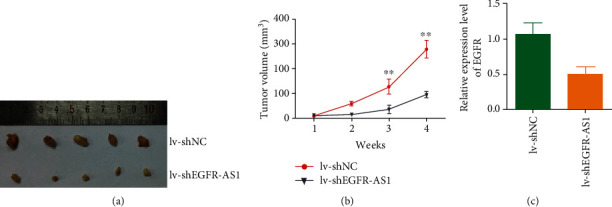
EGFR-AS1 knockdown inhibits bladder cancer growth and metastasis in vivo. (a) Nude mice were given xenografts of EGFR-AS1 knockdown (lv-shEGFR-AS1) and control 5637 cells (5 × 10^6^ cells per site). The xenograft tumors were harvested and photographed after approximately 4 weeks (*n* = 5 per group). (b) The growth curve of EGFR-AS1 knockdown (lv-shEGFR-AS1) tumors compared to control 5637 tumors; bars indicate SD. (c) The expression of EGFR mRNA in the EGFR-AS1 knockdown tumors and control group.

To determine the role of EGFR-AS1 in vivo, EGFR-AS1 knockdown cell line and control group were injected into nude mice. After several weeks of observation, we found that the tumor growth on the side of the injection knockdown EGFR-AS1 was slower (Figures 4(a) and 4(b)). We detected the expression of EGFR mRNA in the transplanted tumor and found that knocking down EGFR-AS1 resulted in lower EGFR mRNA expression (Figure 4(c)). This indicates that EGFR-AS1 can promote the growth and metastasis of bladder cancer in vivo.

### 3.5. EGFR-AS1 Upregulates EGFR mRNA Expression by Maintaining Its Stability

**Figure 5:**
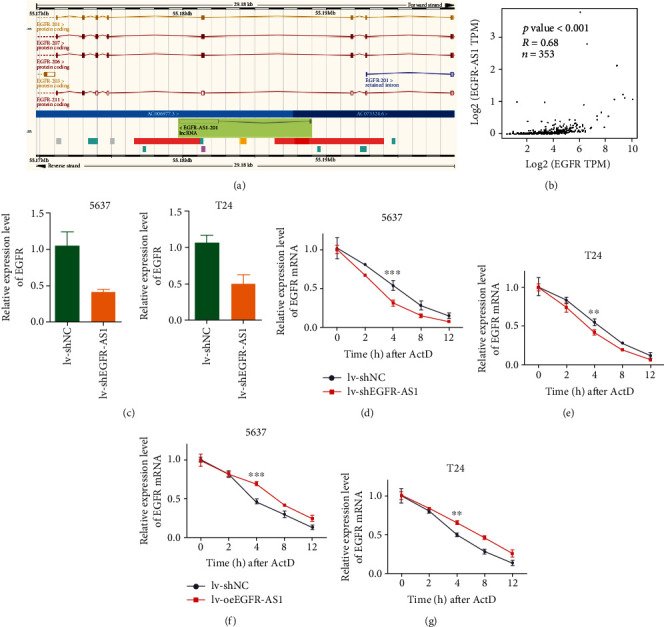
EGFR-AS1 promotes EGFR expression by maintaining its RNA stability. (a) The EGFR-AS1 and EGFR sequences have partial complementarity in the genome. (b) The correlation analysis between the EGFR-AS1 expression and EGFR expression in the TCGA database. (c) Knocking down EGFR-AS1 resulted in lower expression of EGFR mRNA in bladder cancer cells. RNA stability were measured using Actinomycin D. Knocking down EGFR-AS1 decreased the stability of EGFR mRNA in 5637 (d) and T24 cell (e), maximize at 4 hours. Overexpressing EGFR-AS1 enhanced the stability of EGFR mRNA in 5637 (f) and T24 cell (g).

EGFR-AS1 and EGFR have a certain sequence complementarity (Figure 5(a)); thus, we explore the effect of EGFR-AS1 on EGFR expression. The correlation analysis suggested EGFR-AS1 was significantly positively correlated with EGFR using the TCGA database (Figure 5(b)). We found that after knocking down EGFR-AS1, the expression of EGFR mRNA was significantly reduced (Figure 5(c)). We used actinomycin D, a transcription inhibitor, to detect the stability of EGFR mRNA. Similar to renal cancer, knocking down EGFR-AS1 significantly diminished the stability of EGFR mRNA. This effect was most obvious after 4 h (Figures 5(d)–5(g)). Previous experiments have shown that EGFR mRNA can be detected in the EGFR-AS1 pulldown product, indicating that EGFR-AS1 and EGFR have a direct binding effect [6]. These studies demonstrate that in bladder cancer cells, EGFR-AS1 can directly bind to EGFR mRNA and increase its stability.

In conclusion, our research showed that EGFR-AS1 was associated with aggressive clinical features of bladder cancer, and high expression of EGFR-AS1 predicts poor prognosis for bladder cancer patients. EGFR-AS1 enhanced the propagation and invasion of bladder cancer cells in vitro and in vivo. EGFR-AS1 promoted EGFR mRNA expression by maintaining its stability. The EGFR-AS1/EGFR pathway may be developed into diagnostic markers and potential targets of bladder cancer.

## 4. Discussion

LncRNA EGFR-AS1 was selected from big data of renal cancer. We found that EGFR-AS1 promoted renal cancer progression. EGFR-AS1 is closely related to EGFR and is a splicing by-product. Many studies have shown that EGFR played an essential role in bladder cancer [9, 12]. Therefore, we tried to study the role of EGFR-AS1 in bladder cancer. The experiments in vitro and in vivo showed that EGFR-AS1 enhanced the metastatic capacity of bladder cancer cells. Clinical data analysis also demonstrated that EGFR-AS1 was closely related to the aggressive clinical characteristics and poor prognosis of bladder cancer. These studies indicated the promoting effect of EGFR-AS1 on bladder cancer.

Many studies have shown the crucial effect of EGFR on bladder cancer. Gao et al. found that TRIP13 could directly bind to EGFR and regulate the EGFR signaling pathway to promote the formation of bladder cancer [13]. The DDX31/Mutant-p53/EGFR pathway promoted the metastasis ability of muscle invasive bladder cancer [12]. Peng et al.' research showed that metformin and gefitinib could jointly inhibit the growth of bladder cancer through the AMPK and EGFR pathways, providing a new solution for postoperative infusion chemotherapy for bladder cancer [14]. Moreover, similar to renal cancer, our research indicated that EGFR-AS1 could also promote the stability of EGFR mRNA, upregulate EGFR expression, and thereby promote the metastasis of bladder cancer. Tan et al. found that the use of locked nucleic acids to inhibit EGFR-AS1 expression in vivo can inhibit the progression of squamous cell carcinoma [7]. The strategy of targeting EGFR-AS1 based on RNA interference technology has been clinically tested [15]. At present, EGFR inhibitors have not yet been used clinically in bladder cancer. The exploration of their upstream and downstream and joint inhibitors may be a good direction. For example, bladder cancer's growth and metastasis may be repressed by jointly inhibiting the EGFR-AS1/EGFR pathway.

In recent years, immunotherapy has become a new possible direction for the treatment of bladder cancer. We also analyzed the correlation between EGFR-AS1 and immune pathways of bladder cancer and found that EGFR-AS1 was significantly correlated with the TCR signaling pathway and cytokine receptors. The TCR signaling pathway is an important pathway for the human body to play an antitumor effect. The study also found the close relation of EGFR-AS1 and CD8_T cell, indicating that EGFR-AS1 may be responsible for the activation of the immune recognition signaling pathway in bladder cancer.

## 5. Conclusion

In conclusion, our study indicated that EGFR-AS1 enhanced the aggressive characteristics of bladder cancer and predicts poor prognosis. Mechanistically, EGFR-AS1 mainly promotes the expression of EGFR by maintaining the stability of EGFR mRNA and then promotes the metastasis of bladder cancer. Our study indicates that the EGFR-AS1/EGFR pathway can be used as a diagnostic marker and potential target for bladder cancer.

## Figures and Tables

**Table 1:** Correlations between EGFR-AS1 expression and clinicopathological features.

Variables	Low EGFR-AS1 (*n* = 64)	High EGFR-AS1 (*n* = 64)	*p* value
Gender			0.283
Male	40	34	
Female	24	30	
Age			0.592
≤60	35	38	
>60	29	26	
Tumor size (cm)			0.050
≤3 cm	41	30	
>3 cm	23	34	
Pathology stage			0.013^∗^
Ta-T1	43	29	
T2-T3	21	35	
Grade			0.021^∗^
G1-G2	36	23	
G3	28	41	
Lymphatic metastasis			0.023∗
No	54	43	
Yes	10	21	
Recurrence			0.042∗
No	47	36	
Yes	17	28	

^∗^ *p* values < 0.05 were considered statistically significant.

**Table 2:** Correlation analysis between EGFR-AS1 and immune pathways in bladder cancer.

IncRNA symbol	Immune pathway/cell	*p* value	Marker gene number
EGFR-AS1	TCR signaling pathway	0.002^∗^	20
EGFR-AS1	Cytokine receptors	0.006^∗^	66
EGFR-AS1	BCR signaling pathway	0.06	11
EGFR-AS1	Antigen processing and presentation	0.079	25
EGFR-AS1	Interleukin receptor	0.079	19
EGFR-AS1	Natural killer cell cytotoxicity	0.092	14
EGFR-AS1	CD8_Tcell	0.025^∗^	/
EGFR-AS1	Neutrophil	0.136	/
EGFR-AS1	Dendritic	0.135	/
EGFR-AS1	B_cell	0.359	/
EGFR-AS1	CD4_Tcell	0.409	/
EGFR-AS1	Macrophage	0.855	/

^∗^ *p* values < 0.05 were considered statistically significant.

## Data Availability

The data used to support the findings of this study are included within the article.
